# Close co-variation between soil moisture and runoff emerging from multi-catchment data across Europe

**DOI:** 10.1038/s41598-020-61621-y

**Published:** 2020-03-16

**Authors:** Navid Ghajarnia, Zahra Kalantari, René Orth, Georgia Destouni

**Affiliations:** 1 Department of Physical Geography, Bolin Centre for Climate Research, Stockholm University, SE-10691, Stockholm, Sweden; 2 Department of Biogeochemical Integration, Max Planck Institute for Biogeochemistry, D-07745, Jena, Germany

**Keywords:** Hydrology, Hydrology

## Abstract

Soil moisture is an important variable for land-climate and hydrological interactions. To investigate emergent large-scale, long-term interactions between soil moisture and other key hydro-climatic variables (precipitation, actual evapotranspiration, runoff, temperature), we analyze monthly values and anomalies of these variables in 1378 hydrological catchments across Europe over the period 1980–2010. The study distinguishes results for the main European climate regions, and tests how sensitive or robust they are to the use of three alternative observational and re-analysis datasets. Robustly across the European climates and datasets, monthly soil moisture anomalies correlate well with runoff anomalies, and extreme soil moisture and runoff values also largely co-occur. For precipitation, evapotranspiration, and temperature, anomaly correlation and extreme value co-occurrence with soil moisture are overall lower than for runoff. The runoff results indicate a possible new approach to assessing variability and change of large-scale soil moisture conditions by use of long-term time series of monitored catchment-integrating stream discharges.

## Introduction

**Figure 1:**
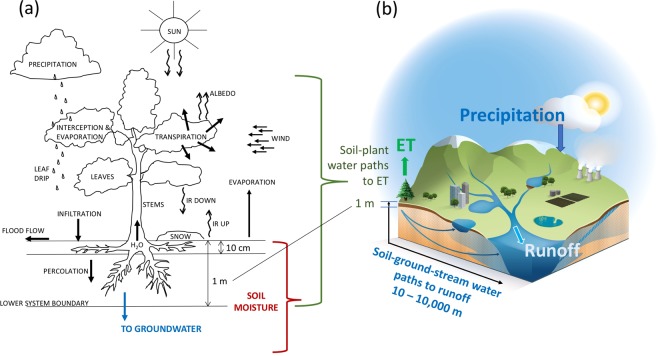
Schematic conceptualization of (**a**) the near-surface soil-plant water system feeding actual evapotranspiration (ET), and (**b**) the full surface and subsurface hydrological catchment system feeding both ET and runoff/stream discharge. The conceptualization perspective (**a**) includes the local near-surface hydrological pathways through soil moisture to ET, as commonly considered and, by certain assumptions, scaled up in large-scale land surface schemes of Earth System Models (ESMs) and surface-focused hydrological modelling. The conceptualization perspective (**b**) includes both the near-surface interaction pathways between catchment-average precipitation, soil moisture and ET, and the mainly subsurface soil-ground-stream water pathways to catchment-average runoff that feeds the total stream discharge from each catchment of any spatial scale. Panel (**b**) represents the conceptualization and interactions considered and quantified in this study; it is redrawn from Supplementary Figure 8 in^5^. The scheme in panel (**a**) represents the hydrological conceptualization proposed in^37^ and is redrawn from the corresponding Figure 6–5 in^38^.

Soil moisture is recognized as an important variable for land-climate interactions and extreme events^1–3^ as well as for hydrology and its extremes in the landscape^4,5^. For quantification of this variable, focus has been largely concentrated on the near-surface domain (Fig. 1a), for example in assessments of soil moisture variability over the landscape^6^ and in land-surface schemes of Earth System Models (ESMs)^7^. However, the depth-extent of the vadose zone down to the groundwater table, over which soil moisture (water content, degree of saturation) varies in time, is in itself a dynamic variable^8^. In this variable vadose zone extent, soil moisture variations interact with precipitation and actual evapotranspiration (ET), and these near-surface variations are commonly accounted for in ESMs and surface-focused hydrological modelling^9^ (Fig. 1a). However, along the subsurface water pathways (Fig. 1b), soil moisture also interacts with the groundwater table depth and its variations^8^, and further with the runoff and stream discharge generation^10^ that is fed by the variable groundwater flow (as driven by the groundwater level variations) over each hydrological catchment.

The complex interactions and degrees of co-variation of soil moisture with precipitation, ET and runoff, through groundwater changes, are often insufficiently represented in large-scale modelling, e.g., by ESMs^7^, and remain largely unresolved for different climate conditions^4,11^. Furthermore, also hydrological models often perform poorly under changing conditions^12–14^, which include the change interactions of soil moisture with vegetation and ET (Fig. 1a) as well as with groundwater^8^ and runoff^10^ (Fig. 1b). Regarding the latter, however, surface-focused modelling only considers a single term of water “loss” to groundwater (blue in Fig. 1a).

From a scientific perspective, it is essential to know the degrees of soil moisture interactions with both ET and runoff in order to answer several key open questions identified by the hydrological community^15^. These include for example: (i) if the hydrological cycle is regionally accelerating/decelerating under climate and environmental change (problem number 1 of 23 community-formulated unsolved problems in hydrology^15^); (ii) how land cover change and soil disturbances impact on water fluxes at the land surface (problem 4); (iii) what causes spatial variations in runoff, evaporation, and subsurface water fluxes (problem 5); (iv) what the hydrologic laws are at the catchment scale and how they change with scale (problem 6); (v) how flood-rich and drought-rich periods arise and are changing (problem 9); (vi) how innovative technologies (e.g., satellite and re-analysis products) can be used to quantify hydrological states and fluxes at a range of spatial and temporal scales (problem 16). From a practical management perspective, it is also essential to know if soil moisture responses to precipitation changes feed mostly into ET (through vegetation) or runoff (through groundwater) changes. Dominant propagation to ET changes may imply criticality for vegetation and crop security^16^, while dominant propagation to runoff changes may imply criticality for freshwater^17^ and energy^18^ security, flood risk^8,19,20^, irrigation timing during droughts^5^, and tradeoffs between future water storage needs^21^ and their hydro-climatic feedbacks^22,23^.

Advancing our quantitative understanding of soil moisture interaction strengths with different hydrological fluxes, towards answering the above open questions, requires comparison and benchmarking of recent/current relationships between soil moisture and flux anomalies over whole catchments (Fig. 1b) and not just their local or upscaled surface conditions (Fig. 1a). Furthermore, the recent hydrological community synthesis of such key questions^15^ has emphasized data-driven multi-catchment comparisons^5,23,24^ as a main method for building up hydrological knowledge that goes beyond and can bridge a fragmented understanding of local inter-compartment fluxes.

This study uses such a multi-catchment comparative approach to quantify possible dominant interactions emerging at large regional scales from available long-term catchment data. A main hypothesis addressed and tested in the study is that dominant soil moisture co-variation with runoff may emerge as a clear large-scale statistical signal^5,10,19^, above local differences in hydro-climate and human activities prevailing in catchments of different scales and in different parts of the world. The motivation for this hypothesis is twofold. First, strong soil moisture correlation with runoff has been indicated by recent results of multi-catchment data analyses for droughts^5^ and floods^19^, and multi-catchment model-data comparisons showing good agreement of modelled catchment-average soil moisture based on runoff data with remotely sensed large-scale soil moisture^10^. Second, if this hypothesis were supported by more multi-catchment data in different world regions, it would open a new avenue of using long-term time series of monitored catchment-integrating stream discharges to assess related variations of large-scale soil moisture conditions^25^, complementing other estimates and interpretations of the latter from upscaled locally measured data and remotely sensed data. A main aim of this study is to further investigate this complementary possibility by evaluating and comparing data-given statistical co-variation patterns between catchment-average soil moisture, runoff and other hydro-climatic variables in and across multiple study catchments.

**Figure 2:**
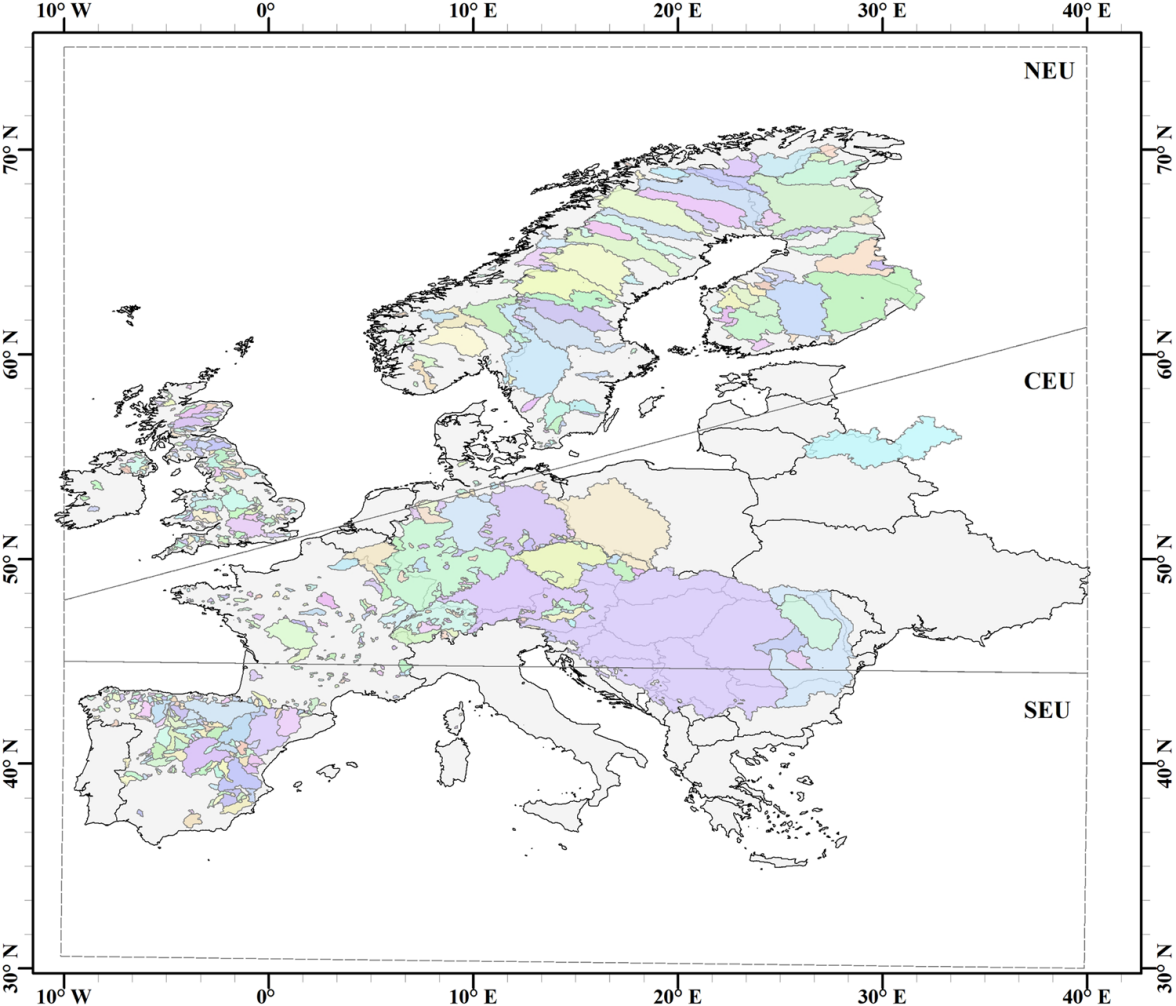
Location of the 1378 study catchments in the three IPCC-determined distinct European climate regions: North Europe (NEU), Central Europe (CEU), and Southern Europe (SEU). Catchments are shown in color and countries in grey.

To achieve this aim, we compile and use three alternative datasets from global observation and re-analysis products covering at least the 30-year period 1980–2010 (Table 1; further data descriptions and references in Methods) for 1378 catchments across Europe (Fig. 2). The study focus on this time period and Europe is due to the required good long-term multi-variable data availability for these catchments across this continent over this study period. Europe also spans steep climate gradients, such that we can study the targeted possible emergent large-scale patterns of soil moisture co-variation with other hydro-climatic variables in and across the three main European climate regions distinguished and proposed by the Intergovernmental Panel on Climate Change (IPCC) as appropriate for managing risks of extreme events and advancing climate change adaptation^2^: Northern Europe (NEU), Central Europe (CEU), and Southern Europe (SEU). Recent large-scale multi-catchment studies of current^5^ and past^26^ hydro-climate across Europe have also shown distinctly different large-scale characteristics of these three climate zones with regard to soil moisture anomaly responses to atmospheric driver variations. To also assess data uncertainty effects, the analysis combines the three alternative data products into a fully independent set for a basic study, as well as an internally consistent and an intermediate dataset for comparative study (Table 1).

**Table 1:** The data products and three dataset combinations used in the study.

Variable	Data product	Included in dataset	Temporal extent	Spatial resolution
Evapo-transpiration (actual)	ERA-Interim/Land reanalysis^a^	Internally consistent	1979–2010	0.25° × 0.25°
	GLEAM-3.2a model^b^	Intermediate & Fully independent	1980–2017	0.25° × 0.25°
Precipitation	ERA-Interim reanalysis^c^	Internally consistent	1979–2018	0.25° × 0.25°
	GPCC-V7^d^	Intermediate & Fully independent	1901–2013	0.5° × 0.5°
Soil moisture	ERA-Interim/Land reanalysis^a^	Internally consistent & Fully independent	1979–2010	0.25° × 0.25°
	GLEAM-3.2a model^b^	Intermediate	1980–2017	0.25° × 0.25°
Temperature	ERA-Interim reanalysis^c^	Internally consistent	1979–2018	0.25° × 0.25°
	GHCN_CAMS^e^	Intermediate & Fully independent	1948–2018	0.5° × 0.5°
Runoff	GSIM^f^	Internally consistent & Intermediate & Fully independent	1901–2012	Catchment-based

^a^European centre for medium-range weather forecasts (ECMWF) Re-Analysis (ERA)-Interim/Land reanalysis datasets^33,34^.

^b^Global Land Evaporation Amsterdam Model (GLEAM-3.2a)^35,36^.

^c^ERA-Interim re-analysis dataset^27^.

^d^Global Precipitation Climatology Centre-Version7 (GPCC-V7)^28,29^.

^e^Global Historical Climatology Network-Climate Anomaly Monitoring System (GHCN_CAMS)^30^.

^f^Global Streamflow Indices and Metadata (GSIM) archive^31,32^.

## Results

**Figure 3:**
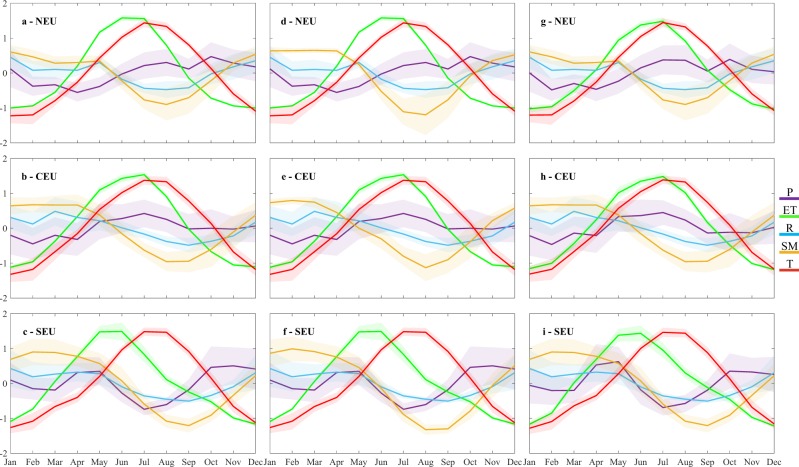
Normalized long-term average monthly variations ${Nor}\overline{{{X}}_{{m}}}$ according to Eq. (1). for precipitation (P), evapotranspiration (ET), runoff (R), soil moisture (SM), and surface temperature (T) in: (**a,d,g**) Northern Europe (NEU), (**b,e,h**) Central Europe (CEU), and (**c**,**f**,**i**) Southern Europe (SEU). Graphs on the left (**a–c**), middle (**d–f**), and right (**g–i**) are for the fully independent, intermediate, and internally consistent datasets (Table 1), respectively. Shadows show interquartile ranges around the normalized long-term average monthly values of each variable.

Aggregating the data for each hydro-climatic variable to catchment scale and further to climate region (see Methods), long-term average variable values ($\overline{{{X}}_{{m}}}$) for each month in the average year can be directly compared through normalization as:

1 $${Nor}\overline{{{X}}_{{m}}}=\frac{\overline{{{X}}_{{m}}}-\bar{{X}}}{{{\sigma }}_{{X}}}$$

where $\bar{{X}}$ and *σ*_*x*_ are the month-independent long-term average value and the standard deviation, respectively, of each variable *X* over all months in the study period 1980–2010. Equation(1) thus normalizes the long-term average value ($\overline{{{X}}_{{m}}}$) of each variable for each month of the average year to multi-variable comparable values of ${Nor}\overline{{{X}}_{{m}}}$. Figure 3 shows the normalized long-term average monthly variations of each variable *X* (solid lines) calculated by applying Eq. (1) on the aggregated time series of *X* over each region. The shadows in Fig. 3 show the inter-quartile range (IQR) of *X* values for each month of the year over the whole time period of analysis (30 values for each month from 1980 to 2010), as a measure of *X* variability around its average value for each month of the year. By plotting ${Nor}\overline{{{X}}_{{m}}}$ for the different variables *X* in Fig. 3, we can see the average monthly variation of each *X* over the average year, along with the corresponding monthly IQR of each *X* for each month among the different years in the study period. This allows direct comparison and identification of possible time lags in the variations of the different monthly averages, and their fluctuations around those averages among different years.

Comparison of resulting ${Nor}\overline{{{X}}_{{m}}}$ for the different variables (Fig. 3) shows that the soil moisture variations (yellow) are most consistent with the runoff variations (light blue) across all three European climate regions. In contrast, the ET variations (green) are most consistent with the temperature variations (red), and the variations of both these variables differ considerably from the soil moisture and runoff variations. The monthly precipitation variations (dark blue) are overall smaller than and differ from those of the other hydro-climatic variables while its IQR is wider than for all other variables. Temperature and ET have overall the smallest IQR in all three climate regions, but the variation range of ET is higher in the drier SEU region than in the wetter CEU and NEU regions. The IQRs of soil moisture and runoff are intermediate and are smallest during the driest months (Aug and Sep) of the relatively dry SEU region.

The peaks in average monthly ET and temperature, which both occur in summer, exhibit a time lag (with ET peaking and starting to decline before temperature) that is greater in SEU than in NEU and CEU (Fig. 3). This lag depends on the corresponding average variations in monthly soil moisture and precipitation, which together provide the water availability for ET in each month. Across all climate regions, soil moisture declines considerably from around May and remains relatively low during summer. In SEU, precipitation also declines and remains relatively low during summer, while it remains relatively high throughout summer in NEU and CEU. A relatively high water supply from precipitation is thus available for ET (even though soil moisture declines) through the summer months in NEU and CEU. Nevertheless, ET in NEU and CEU declines while precipitation is still increasing (in NEU) or remains relatively high (in CEU) after the decrease in temperature during Jul, Aug, and Sep. This ET decline, while precipitation remains high, reflects that average monthly ET is here energy limited; as such, ET in NEU and CEU can only efficiently use the relatively high water availability in the warm months from start to end of summer, but not thereafter when temperature and thus energy supply declines while water availability is still relatively high. In SEU, however, both soil moisture and precipitation are relatively small during summer, reflecting water limitation of ET in this region; ET therefore starts here to decline already around June, as the water provided from precipitation becomes then insufficient for keeping up a high ET level, even though the temperature and associated energy supply remain high until later in the average year. These results for ${Nor}\overline{{{X}}_{{m}}}$ are robust for the fully independent, intermediate, and internally consistent datasets (left, middle, and right, respectively, Fig. 3).

The whole time series of variable *X* values in each month *m* of each year *y* over the whole study period 1980–2010 is further compared for all data products in terms of absolute monthly values (*X*_*m,y*_; Supporting Figures S1–S4) and corresponding normalized anomalies (*NorX*_*m,y*_; Supporting Figures S5–S8) calculated as:

2 $${Nor}{{X}}_{{m},{y}}=\frac{{{X}}_{{m},{y}}-\overline{{{X}}_{{m}}}}{{{\sigma }}_{{{X}}_{{m}}}}$$

where $\overline{{{X}}_{{m}}}$ and ${{\sigma }}_{{{X}}_{{m}}}$ are the long-term average value and standard deviation of monthly *X*, respectively, for each month *m* over all years in the study period 1980–2010. The comparisons of monthly absolute and anomaly values of *X* show high level of agreement between the variables, regardless of which data product they belong to, with an exception for the absolute *X*_*m,y*_ values of soil moisture; for this variable, the ERA-Interim/Land reanalysis product (used in the fully independent and internally consistent datasets) has overall higher mean values and smaller variations than the GLEAM-3.2a product (used in the intermediate dataset) (Figure S3). The differences in absolute soil moisture are greatest for NEU, smaller for CEU, and smallest for SEU, while corresponding differences all disappear when comparing the normalized anomalies *NorX*_*m,y*_ for soil moisture (Figure S7).

**Figure 4:**
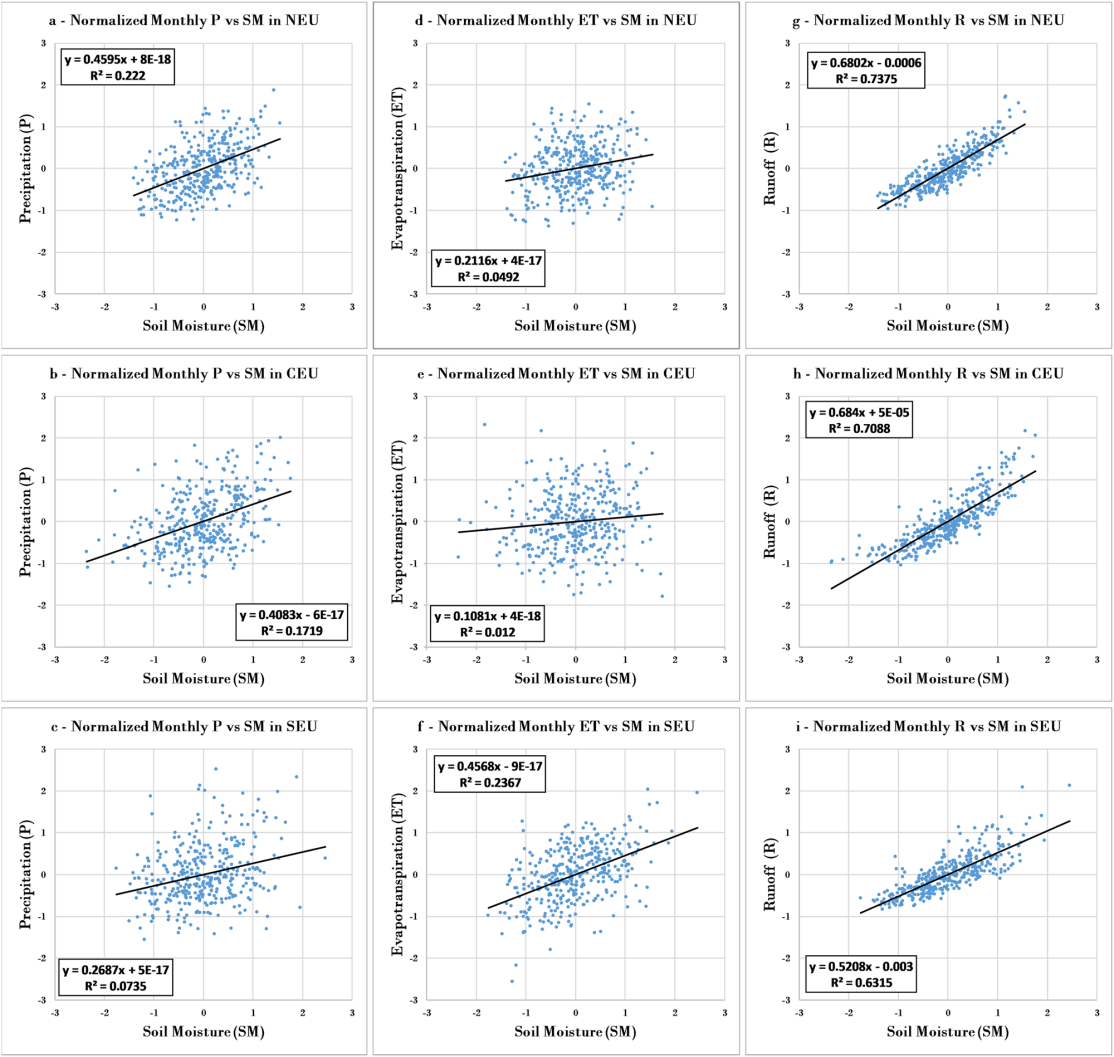
Scatter plots and regression lines for normalized monthly anomalies, *NorX*_*m,y*_ according to Eq. (2), of precipitation (P, left), evapotranspiration (ET, middle), and runoff (R, right) versus soil moisture (SM) for (**a,d,g**) Northern Europe (NEU), (**b,e,h**) Central Europe (CEU), and (**c,f,i**) Southern Europe (SEU). The results shown here are for the fully independent dataset (Table 1).

Figure 4 shows scatter plots and regression lines for the normalized anomalies *NorX*_*m,y*_ of precipitation (left panels), ET (middle panels) and runoff (right panels) versus those of soil moisture based on the fully independent dataset. Supporting Figure S9 shows corresponding results from this dataset also for temperature versus soil moisture anomalies (exhibiting no correlation), while Supporting Figures S10-S11 show *NorX*_*m,y*_ results for all variables based on the intermediate and internally consistent datasets, respectively. The normalized soil moisture and runoff anomalies exhibit by far the greatest correlations across all three climate regions and based on all three datasets (Fig. 4 and Supporting Figures S10-S11). Furthermore, when comparing the points for the most dry and wet soil moisture conditions within the dataset, the same data points also largely represent the most dry and wet runoff conditions, respectively (right panels, Fig. 4). This indicates largely concurrent occurrences of extreme soil moisture and runoff conditions. In contrast, precipitation (left panels) and ET (middle panels) vary over a large range, from negative to positive values, during both the driest (less than −1.5 normalized monthly values) and the wettest (greater than +1.5 normalized monthly values) data points for soil moisture. This indicates largely uncorrelated extreme occurrences between soil moisture and precipitation or ET. These correlation and extreme co-occurrence results are robust also when using the intermediate and internally consistent datasets (Supporting Figures 10–11).

Note that Fig. 3 shows the intra-annual variation of long-term average monthly values (${Nor}\overline{{{X}}_{{m}}}$, Eq. (1)), while Fig. 4 shows the *X* anomalies for each month of each year from these average conditions in each month of the average year *NorX*_*m,y*_, Eq. (2)). As such, the correlation patterns are not and should not be expected to be the same for these different quantities, ${Nor}\overline{{{X}}_{{m}}}$ and *NorX*_*m,y*_. For example, the average seasonal variability within each year quantified by ${Nor}\overline{{{X}}_{{m}}}$ shows negative correlation between ET and soil moisture because ET is on average largest in the warm season when it is also driest and soil moisture is on average the smallest (Fig. 3). In contrast, the monthly anomalies from this average seasonal variation exhibit positive correlation between ET and soil moisture because the individual values of these variables in some month within the time series are both similarly affected if this month is anomalously wet or dry relative to the long-term average seasonal conditions for that specific month order within the average year (Fig. 4).

**Figure 5:**
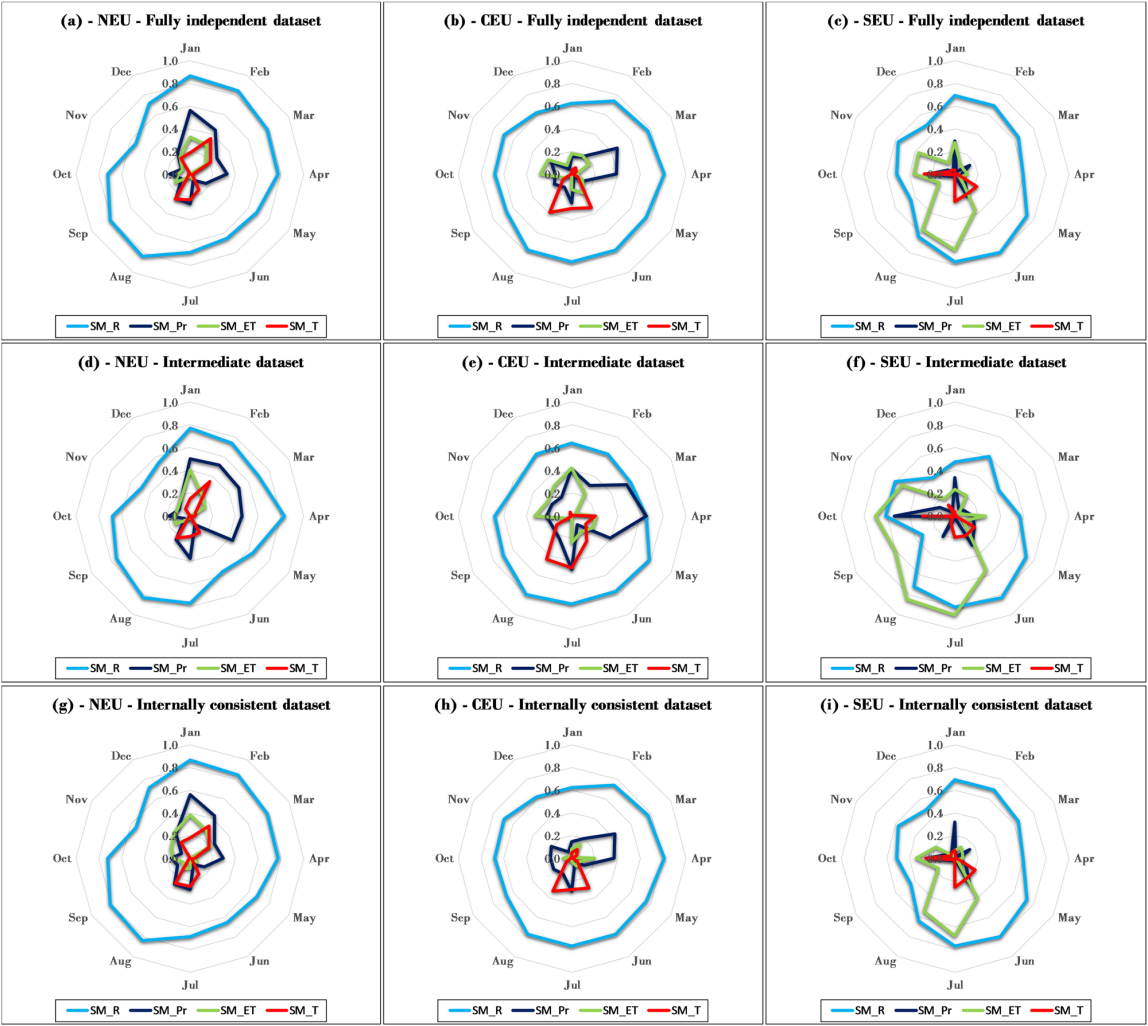
Monthly coefficient of determination (r^2^) between normalized anomalies, *NorX*_*m,y*_ according to Eq. (2), of the monthly values of runoff (R), precipitation (P), evapotranspiration (ET) and temperature versus those of soil moisture (SM) in (**a,d,g**) North Europe (NEU), (**b,e,h**) Central Europe (CEU), and (**c,f,i**) Southern Europe (SEU). The r^2^ values summarize the results of linear regressions shown in Supporting Figures S12-S23.

For further distinction of seasonal co-variations, Fig. 5 summarizes all monthly coefficients of determination (r^2^) between the normalized monthly anomalies *NorX*_*m,y*_ of soil moisture and those of the other hydro-climatic variables for the NEU, CEU, and SEU regions and for all of the fully independent, intermediate, and internally consistent datasets. Supporting Figures S12-S15 (fully independent dataset), S16-S19 (intermediate dataset), and S20-S23 (internally consistent dataset) further show the underlying monthly scatter plots and regression lines for the anomalies *NorX*_*m,y*_ of precipitation (Figures S12, S16, and S20), ET (Figures S13, S17, and S21), runoff (Figures S14, S18, and S22) and temperature (Figures S15, S19, and S23) versus that of soil moisture. For runoff and soil moisture, their monthly anomaly correlations are overall relatively high (Fig. 5) and their extreme (most dry/wet) monthly values tend to largely co-occur (Supporting Figures S14, S18, and S22); as for their total correlation (Fig. 4), these monthly results are robust across the months and climate regions and for all three datasets. For ET and precipitation versus soil moisture, some regional monthly correlations approach those for runoff, including also relatively consistent co-occurrences of dry/wet events; such region- and month-specific results are more evident in the intermediate than the fully independent or internally consistent datasets. For ET, there is relatively high correlation with soil moisture in the SEU region for the summer to early autumn months of July-October (Fig. 5, Supporting Figures S13, S17, and S21). For precipitation, some relatively high correlations appear for some winter/spring months during January-April in the NEU and CEU regions (Fig. 5, Supporting Figures S12, S16, and S20). For temperature versus soil moisture, monthly correlations are generally low (Fig. 5), or inconsistently varying between negative and positive among different months (Supporting Figures S15, S19, and S23).

**Figure 6:**
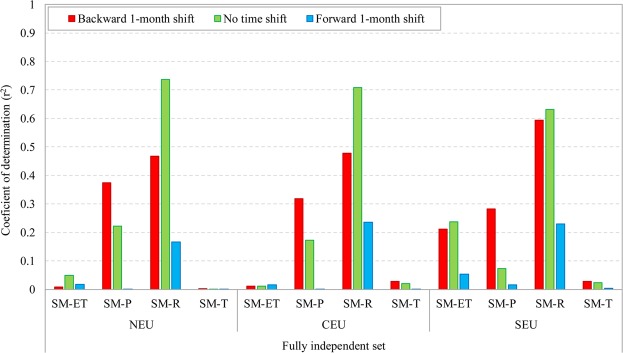
Coefficient of determination (r^2^) between normalized monthly anomalies, *NorX*_*m,y*_ according to Eq. (2), of evapotranspiration (ET), precipitation (P), runoff (R), and temperature (T) versus those of soil moisture (SM) with consideration of a backward 1-month shift (red), no time shift (green), and a forward 1-month shift (blue) between the variables. Results are presented for North Europe (NEU), Central Europe (CEU), and Southern Europe (SEU) based on the fully independent dataset.

The results shown in Figs. 4–5 (and corresponding Supporting Figures in the Supplementary Information) are derived without consideration of possible time lags in the co-variation of the studied variables. To resolve the influence of such time lags, we have also considered temporal shifts in additional analysis of the variable time series (Fig. 6, Supporting Figure S24-S25). Specifically, variable correlations are calculated and compared with the corresponding no-shift results for a backward 1-month shift and a forward 1-month shift of precipitation, ET, runoff, or temperature versus soil moisture. Although some of the studied temporal lag scenarios may not reflect reasonable physical relationships (e.g., the forward temporal shift of precipitation or temperature in relation to soil moisture), the analysis has been systematic and not presuming to know a priori what different temporally shifted co-variation patterns may emerge at large scales. As such, and for illustration consistency and clarity, we have quantified and present all different time lag scenario results.

Figure 6 summarizes these results in terms of their respective r^2^ values, showing that a backward 1-month shift of precipitation versus soil moisture yields higher r^2^ value between these variables than the corresponding no-shift results for all cases except for the NEU and CEU regions based on the fully independent dataset (for the intermediate and internally consistent datasets, see Supporting Figure S24). A marginal increase of r^2^ values relative to the no-shift results can also be seen for a forward 1-month shift of ET versus soil moisture for the CEU region (for all datasets) as well as for a backward 1-month shift of temperature versus soil moisture in most cases. However, no-shift scenario results yield consistently the highest r^2^ value for the correlation between runoff and soil moisture. Importantly, the correlation between runoff and soil moisture also remains the overall highest one among all time lag scenarios (backward, forward, and no shift), across all regions and for all datasets. The obtained co-variation patterns across time lag scenarios are thus consistent with the basic no-shift results in this respect, which strengthens the main finding of a dominant co-variation pattern between soil moisture and runoff (Fig. 6 and Supporting Figure S24; see also Supporting Figure S25, showing the highest monthly r^2^ values from the results of all considered time shifts).

## Discussion

The results of considerably closer co-variation and higher anomaly correlation between runoff (than between precipitation, evapotranspiration, or temperature) and soil moisture may be considered surprising as, e.g., the land surface schemes of ESMs as well as much hydrological modelling have focused primarily on soil moisture interactions with precipitation and ET^7^ (Fig. 1a). However, indications of close relationships between variations^10^ and drought event co-occurrences^5^ of soil moisture and runoff have also emerged from other recent data-driven studies of long-term time series for multiple catchments in Europe and other parts of the world.

Clearly, precipitation, ET, runoff and water storage changes (including soil moisture changes as well as changes in groundwater and surface water levels) are all mechanistically linked through fundamental water balance in the landscape. The present data-given co-variation and correlation results identify some dominant, first-order relationships emerging on large (multi)catchment/regional scales from all the complex local process links and interactions taking place in underlying smaller scales. This emergence indicates a key role for soil moisture in regulating large-scale runoff variations, consistently across European climate regions and datasets. Mechanistically, this role is explainable by soil moisture changes implying corresponding changes in groundwater table depth^8^ and thereby also in the hydraulic gradients that govern groundwater flows towards and into nearest streams (Fig. 1b); these hydraulic-hydrological links apply and emerge as dominant across all studied hydro-climatic conditions. In contrast, ET variations are regulated by temperature variations as long as there is sufficient water supply from precipitation and/or soil moisture to efficiently use the associated energy supply in vegetation transpiration and evaporation. In SEU, where both soil moisture and precipitation are on average small and declining in summer (Fig. 3), water limitation leads to declining ET and thereby to relatively high region- and summer-specific correlation between ET and soil moisture (Fig. 5).

The emergent large-scale relationships found in this multi-catchment study may not be evident in each individual catchment, as various underlying physical processes affect local hydrological behavior in each catchment (e.g. various geological and climatological conditions, anthropogenic influences, different hydrological regime types, and other specific catchment features). However, the fact that a clear statistical large-scale signal of dominant correlation between soil moisture and runoff emerges consistently by use of different datasets from different data sources for numerous catchments with different local features, e.g., including stronger or weaker anthropogenic influences, and annual, seasonal and monthly variation conditions within each climate region, and even with or without consideration of time lags between variables, indicates the main study conclusions as robust and reliable for the European continent and its main climate regions.

## Conclusion

The results of this study show that monthly soil moisture anomalies correlate well with runoff anomalies across multiple study catchments of various scales in the different climate regions of Europe. Average monthly soil moisture and runoff further co-vary, while extreme soil moisture and runoff values also co-occur across Europe. For the other studied hydro-climatic variables (precipitation, ET, temperature), the degrees of co-variation, anomaly correlation and extreme value co-occurrence with soil moisture are predominantly low in the European climates. A summer correlation exception appears in SEU, between the region-specific water-limited and declining ET and the generally declining soil moisture during summer.

As outlined in the Introduction, the results and findings of this study can contribute to efforts for eventually answering a number of community-formulated key open questions in hydrology^15^. Overall, this data-driven multi-catchment and multi-climate study shows robust and mechanistically explainable emergence of dominant, first-order co-variation between soil moisture and runoff on large scales, across the European climates and used datasets. These robust results should be further considered in large-scale soil moisture modeling, for example in the land surface schemes of ESMs and hydrological soil moisture models. The runoff results indicate a possible new, complementary approach to assessing variability and change of large-scale soil moisture conditions by use of long-term time series of monitored catchment-integrating stream discharges. They benchmark a data-given large-scale co-variation pattern that can be used for model testing, but also calls for further research on large-scale pattern emergence in other parts of the world. Moreover, the found soil moisture relationships should also be investigated at finer scales, with explicit consideration of various local factors, such as different catchment features, hydrological regimes, anthropogenic influences, and their influence on the large-scale co-variation patterns.

## Methods

Table 1 lists the data products, and their temporal extents and spatial resolutions, used to analyze the large-scale variation patterns of soil moisture and other main hydro-climatic variables (precipitation, actual ET, runoff and temperature) in the 1378 study catchments across Europe during 1980–2010. Both station-based observation data (the GSIM, GHCN_CAMS and GPCC-V7 products in Table 1) and model-based re-analysis data (the ERA-Interim and GLEAM-3.2a products in Table 1) are used for the different study variables. There is no doubt that the best way to study co-variation patterns would be to use a purely observation-based dataset. However, there is no such database available for soil moisture and ET with homogeneous and consistent temporal and spatial resolution. Therefore, there is an inevitable need to use alternative datasets for soil moisture and ET in this study. For this purpose, we have used model-based re-analysis data, which many researchers regard as the available datasets closest to direct observations^27^. The basic dataset for this study called as the fully independent dataset, includes all available observation-based products for precipitation, temperature, and runoff from GPCC-V7^28,29^, GHCN_CAMS^30^, and GSIM^31,32^ databases, while obtaining soil moisture and ET from two different re-analysis products of ERA-Interim Land re-analysis^33,34^ and GLEAM-3.2a^35,36^, respectively. Conversely, for the variables besides runoff, the internally consistent dataset retrieves all variables from the same re-analysis product (ERA-Interim re-analysis data^27^ and the Land re-analysis data^33,34^), while the intermediate dataset uses soil moisture (which is a key variable in this study) from a different product (GLEAM-3.2a) than the other datasets (ERA-Interim\Land re-analysis data) with keeping the source for other variables the same as the fully independent dataset.

It is worth mentioning that if all variable data are from the same product, there is a risk of discovering relationships built into that re-analysis model. However, by using an independent dataset, there is another risk of discovering unrealistic or disturbed relationships due to possible inconsistencies between the modeling and data assimilation methods used in different re-analysis datasets. In other words, there is always some risk of arriving at misleading correlations based on either an internally consistent or a fully independent dataset, and only comparison with corresponding results from a fully observation-based dataset for all variables (which is not available at the current time for soil moisture and ET) can reveal which type of model-based dataset provides the most realistic results. In the absence of a fully observation-based dataset, we have repeated the analysis for different datasets in order to reveal and evaluate how results depend on the choice of different types of (independent, dependent or intermediate) model-based reanalysis data. The dependency or independency of different datasets or model outputs, however, is not a main or critical investigation issue in this study. We here use and compare results from the multiple datasets to see if the findings of large-scale soil moisture correlations are highly sensitive to (and thereby uncertain) or remain robust irrespective of which specific dataset is used for obtaining these large-scale results.

Among the data for the different study variables (Table 1), the data availability for runoff is limiting, even though the GSIM dataset has greatly expanded this from previous availability of stream discharge data. While data for the other variables are consistently available as global gridded time series with few missing values, the runoff data in GSIM (as in other runoff datasets) represent water flow through the whole catchment of each hydrometric station, with its specific data availability and measurement time series. As such, runoff data (discharge divided by contributing catchment area) is available for 5235 European catchments in the GSIM dataset. However, only 1378 of these catchments have at least 300 non-missing monthly runoff values (corresponding to 25 years) within the study period 1980–2010. This study therefore includes and is based on the relatively complete runoff time series for these 1378 catchments across the three European climate regions of NEU, CEU and SEU (Fig. 2). Many of these catchments may be influenced by human activities and other local conditions affecting their hydrological regimes^23^. However, this study does not aim to study pristine catchments. It aims to test whether a possible large-scale dominance of soil moisture correlation with runoff, or alternatively with ET, or any other of the studied hydro-climatic variables, may emerge as a clear statistical large-scale signal above the noise of all local peculiarities in each of the multiple study catchments.

To get relevant regional statistics for identifying such a possible large-scale signal, all data used in this study are spatially aggregated to catchment scales and then to climate regions. Given the catchment association of runoff data, the datasets for the other hydro-climatic variables also need to be catchment-wise aggregated for consistency and compatibility. Depending on catchment area and grid sizes (spatial resolution) of each dataset, each catchment may include one or more data grid cell(s). The spatial aggregation of the gridded data for each catchment is based on area-weighted catchment averaging over the grid cells covered by the catchment, providing a catchment-specific data time series for each variable. For regional aggregation, the catchment-specific data time series are used for further area-weighted averaging over the study catchments included in each climate region, providing a region-specific data time series for each variable. As for the regional categorization into the three main European climate regions NEU, CEU, and SEU, the study follows the IPCC-proposed delineation of appropriate climate region delineation for risk management of regional extremes and advancement of change adaptation in this continent^2^. These distinct European climate regions also represent a continental-scale climate gradient from the wet and energy limited in NEU, towards the intermediate conditions in CEU, to the relatively dry and water limited conditions in SEU.

## Supplementary information

Supplementary information

## Data Availability

The ECMWF re-analysis for ET and soil moisture from ERA-Interim/Land reanalysis can be found at https://apps.ecmwf.int/datasets/data/interim-land/type=fc/, and the ERA-Interim reanalysis for precipitation and temperature at https://apps.ecmwf.int/datasets/data/interim-full-moda/levtype=sfc/. Soil moisture and ET data from the GLEAM-3.2a model are available at https://www.gleam.eu/. GPCC-V7 precipitation data are available from the NOAA website https://www.esrl.noaa.gov/psd/data/gridded/data.gpcc.html. Temperature data from GHCN_CAMS are available at https://www.esrl.noaa.gov/psd/data/gridded/data.ghcncams.html, and GSIM streamflow and metadata at https://doi.pangaea.de/10.1594/PANGAEA.887470 and https://doi.pangaea.de/10.1594/PANGAEA.887477.
