# Supplementary material for: Close co-variation between soil moisture and runoff emerging from multi-catchment data across Europe
Source: Sci Rep. 2020 Mar 16;10:4817. doi: 10.1038/s41598-020-61621-y (PMC7076032; doi:10.1038/s41598-020-61621-y)
Supplement: Supplementary file 1 — Supplementary information [file 41598_2020_61621_MOESM1_ESM.docx]

Supplementary information for

**Close co-variation between soil moisture and runoff emerging from multi-catchment data across Europe**

Navid Ghajarnia, Zahra Kalantari, René Orth and Georgia Destouni

**Contents**

**Supporting Figures S1-S4** show time series of variable absolute values for all data products

**Supporting Figures S5-S8** show corresponding time series for normalized variable anomalies

**Supporting Figure S9** shows scatter plot of temperature versus soil moisture anomalies for the fully independent dataset

**Supporting Figure S10** shows total scatter plots for all (other: precipitation, evapotranspiration, runoff, and temperature) variable anomalies versus soil moisture anomalies for the intermediate dataset

**Supporting Figure S11** shows total scatter plots for all (other: precipitation, evapotranspiration, runoff, and temperature) variable anomalies versus soil moisture anomalies for the internally consistent dataset

**Supporting Figures S12-S23** show monthly scatter plots for all (other: precipitation, evapotranspiration, runoff, and temperature) variable anomalies versus soil moisture anomalies for the fully independent, intermediate, and internally consistent datasets. Figure (4) in the main paper is obtained from the regression results of r^2^ values in these plots.

**Supporting Figure S24** shows Coefficient of determination (r^2^) between normalized monthly anomalies of evapotranspiration, precipitation, runoff, and temperature versus those of soil moisture with consideration of a backward 1-month shift, no time shift, and a forward 1-month shift between the variables.

**Supporting Figure S25** shows the highest monthly r^2^ values from the results considering different (including no) time shifts of the monthly anomalies of precipitation, evapotranspiration, runoff, or temperature versus those of soil moisture, for all datasets.

|  |
| --- |
|  |
|  |

Figure S1. Long-term monthly variations $\boldsymbol{X}_{\boldsymbol{m,y}}$ for precipitation from 1980 to 2010 in: (a) Northern Europe (NEU), (b) Central Europe (CEU), and (c) Southern Europe (SEU). Blue line is for the GPCC-V7 dataset (used in the intermediate and fully independent datasets), while red line is for the ERA-Interim reanalysis (used in the internally consistent dataset).

|  |
| --- |
|  |
|  |

Figure S2. Long-term monthly variations $\boldsymbol{X}_{\boldsymbol{m,y}}$ for evapotranspiration from 1980 to 2010 in: (a) Northern Europe (NEU), (b) Central Europe (CEU), and (c) Southern Europe (SEU). Blue line is for the GLEAM-3.2a dataset (used in the intermediate and fully independent datasets), while red line is for the ERA-Interim/Land reanalysis (used in the internally consistent dataset).

|  |
| --- |
|  |
|  |

Figure S3. Long-term monthly variations $\boldsymbol{X}_{\boldsymbol{m,y}}$ for soil moisture from 1980 to 2010 in: (a) Northern Europe (NEU), (b) Central Europe (CEU), and (c) Southern Europe (SEU). Blue line is for the GLEAM-3.2a dataset (used in the intermediate and fully independent datasets), while red line is for the ERA-Interim/Land reanalysis (used in the internally consistent dataset).

|  |
| --- |
|  |
|  |

Figure S4. Long-term monthly variations $\boldsymbol{X}_{\boldsymbol{m,y}}$ for temperature from 1980 to 2010 in: (a) Northern Europe (NEU), (b) Central Europe (CEU), and (c) Southern Europe (SEU). Blue line is for the GHCN_CAMS dataset (used in the intermediate and fully independent datasets), while red line is for the ERA-Interim reanalysis (used in the internally consistent dataset).

|  |
| --- |
|  |
|  |

Figure S5. Normalized long-term monthly variations $\boldsymbol{NorX}_{\boldsymbol{m,y}}$ according to Equation (2) for precipitation from 1980 to 2010 in: (a) Northern Europe (NEU), (b) Central Europe (CEU), and (c) Southern Europe (SEU). Blue line is for the GPCC-V7 dataset (used in the intermediate and fully independent datasets), while red line is for the ERA-Interim reanalysis (used in the internally consistent dataset).

|  |
| --- |
|  |
|  |

Figure S6. Normalized long-term monthly variations $\boldsymbol{NorX}_{\boldsymbol{m,y}}$ according to Equation (2) for evapotranspiration from 1980 to 2010 in: (a) Northern Europe (NEU), (b) Central Europe (CEU), and (c) Southern Europe (SEU). Blue line is for the GLEAM-3.2a dataset (used in the intermediate and fully independent datasets), while red line is for the ERA-Interim/Land reanalysis (used in the internally consistent dataset).

|  |
| --- |
|  |
|  |

Figure S7. Normalized long-term monthly variations $\boldsymbol{NorX}_{\boldsymbol{m,y}}$ according to Equation (2) for soil moisture from 1980 to 2010 in: (a) Northern Europe (NEU), (b) Central Europe (CEU), and (c) Southern Europe (SEU). Blue line is for the GLEAM-3.2a dataset (used in the intermediate and fully independent datasets), while red line is for the ERA-Interim/Land reanalysis (used in the internally consistent dataset).

|  |
| --- |
|  |
|  |

Figure S8. Normalized long-term monthly variations $\boldsymbol{NorX}_{\boldsymbol{m,y}}$ according to Equation (2) for temperature from 1980 to 2010 in: (a) Northern Europe (NEU), (b) Central Europe (CEU), and (c) Southern Europe (SEU). Blue line is for the GHCN_CAMS dataset (used in the intermediate and fully independent datasets), while red line is for the ERA-Interim reanalysis (used in the internally consistent dataset).

|  |
| --- |
|  |
|  |

Figure S9. Scatter plots and regression lines for normalized anomalies, $\boldsymbol{NorX}_{\boldsymbol{m}\mathbf{,}\boldsymbol{y}}$ according to Equation (2), of temperature (T) versus soil moisture (SM), for (a) Northern Europe (NEU), (b) Central Europe (CEU), and (c) Southern Europe (SEU) by using the fully independent dataset.

|  |  |  |  |
| --- | --- | --- | --- |
|  |  |  |  |
|  |  |  |  |

**Figure S10.** Scatter plots and regression lines for normalized anomalies, ${NorX}_{m,y}$ according to Equation (2), of precipitation (P, 1^st^ column), evapotranspiration (ET, 2^nd^ column), runoff (R, 3^rd^ column), and temperature (T, 4^th^ column) versus soil moisture (SM) for (a,d,g,j) Northern Europe (NEU), (b,e,h,k) Central Europe (CEU), and (c,f,i,l) Southern Europe (SEU), by using the intermediate dataset.

|  |  |  |  |
| --- | --- | --- | --- |
|  |  |  |  |
|  |  |  |  |

**Figure S11.** Scatter plots and regression lines for normalized anomalies, ${NorX}_{m,y}$ according to Equation (2), of precipitation (P, 1^st^ column), evapotranspiration (ET, 2^nd^ column), runoff (R, 3^rd^ column), and temperature (T, 4^th^ column) versus soil moisture (SM) for (a,d,g,j) Northern Europe (NEU), (b,e,h,k) Central Europe (CEU), and (c,f,i,l) Southern Europe (SEU), by using the internally consistent dataset.

| a – Northern Europe (NEU) | b – Central Europe (CEU) | c – Southern Europe (SEU) |
| --- | --- | --- |
|  |  |  |

**Figure S12.** Scatter plots and regression lines for normalized anomalies, ${NorX}_{m,y}$ according to Equation (2), of precipitation (P, Y-axis) versus soil moisture (SM, X-axis), for (a) Northern Europe (NEU), (b) Central Europe (CEU), and (c) Southern Europe (SEU) by using the fully independent dataset.

| a – Northern Europe (NEU) | b – Central Europe (CEU) | c – Southern Europe (SEU) |
| --- | --- | --- |
|  |  |  |

**Figure S13.** Scatter plots and regression lines for normalized anomalies, ${NorX}_{m,y}$ according to Equation (2), of evapotranspiration (ET, Y-axis) versus soil moisture (SM, X-axis), for (a) Northern Europe (NEU), (b) Central Europe (CEU), and (c) Southern Europe (SEU) by using the fully independent dataset.

| a – Northern Europe (NEU) | b – Central Europe (CEU) | c – Southern Europe (SEU) |
| --- | --- | --- |
|  |  |  |

**Figure S14.** Scatter plots and regression lines for normalized anomalies, ${NorX}_{m,y}$ according to Equation (2), of runoff (R, Y-axis) versus soil moisture (SM, X-axis), for (a) Northern Europe (NEU), (b) Central Europe (CEU), and (c) Southern Europe (SEU) by using the fully independent dataset.

| a – Northern Europe (NEU) | b – Central Europe (CEU) | c – Southern Europe (SEU) |
| --- | --- | --- |
|  |  |  |

**Figure S15.** Scatter plots and regression lines for normalized anomalies, ${NorX}_{m,y}$ according to Equation (2), of temperature (T, Y-axis) versus soil moisture (SM, X-axis), for (a) Northern Europe (NEU), (b) Central Europe (CEU), and (c) Southern Europe (SEU) by using the fully independent dataset.

| a – Northern Europe (NEU) | b – Central Europe (CEU) | c – Southern Europe (SEU) |
| --- | --- | --- |
|  |  |  |

**Figure S16.** Scatter plots and regression lines for normalized anomalies, ${NorX}_{m,y}$ according to Equation (2), of precipitation (P, Y-axis) versus soil moisture (SM, X-axis), for (a) Northern Europe (NEU), (b) Central Europe (CEU), and (c) Southern Europe (SEU) by using the intermediate dataset.

| a – Northern Europe (NEU) | b – Central Europe (CEU) | c – Southern Europe (SEU) |
| --- | --- | --- |
|  |  |  |

**Figure S17.** Scatter plots and regression lines for normalized anomalies, ${NorX}_{m,y}$ according to Equation (2), of evapotranspiration (ET, Y-axis) versus soil moisture (SM, X-axis), for (a) Northern Europe (NEU), (b) Central Europe (CEU), and (c) Southern Europe (SEU) by using the intermediate dataset.

| a – Northern Europe (NEU) | b – Central Europe (CEU) | c – Southern Europe (SEU) |
| --- | --- | --- |
|  |  |  |

**Figure S18.** Scatter plots and regression lines for normalized anomalies, ${NorX}_{m,y}$ according to Equation (2), of runoff (R, Y-axis) versus soil moisture (SM, X-axis), for (a) Northern Europe (NEU), (b) Central Europe (CEU), and (c) Southern Europe (SEU) by using the intermediate dataset.

| a – Northern Europe (NEU) | b – Central Europe (CEU) | c – Southern Europe (SEU) |
| --- | --- | --- |
|  |  |  |

**Figure S19.** Scatter plots and regression lines for normalized anomalies, ${NorX}_{m,y}$ according to Equation (2), of temperature (T, Y-axis) versus soil moisture (SM, X-axis), for (a) Northern Europe (NEU), (b) Central Europe (CEU), and (c) Southern Europe (SEU) by using the intermediate dataset.

| a – Northern Europe (NEU) | b – Central Europe (CEU) | c – Southern Europe (SEU) |
| --- | --- | --- |
|  |  |  |

**Figure S20.** Scatter plots and regression lines for normalized anomalies, ${NorX}_{m,y}$ according to Equation (2), of precipitation (P, Y-axis) versus soil moisture (SM, X-axis), for (a) Northern Europe (NEU), (b) Central Europe (CEU), and (c) Southern Europe (SEU) by using the internally consistent dataset.

| a – Northern Europe (NEU) | b – Central Europe (CEU) | c – Southern Europe (SEU) |
| --- | --- | --- |
|  |  |  |

**Figure S21.** Scatter plots and regression lines for normalized anomalies, ${NorX}_{m,y}$ according to Equation (2), of evapotranspiration (ET, Y-axis) versus soil moisture (SM, X-axis), for (a) Northern Europe (NEU), (b) Central Europe (CEU), and (c) Southern Europe (SEU) by using the internally consistent dataset.

| a – Northern Europe (NEU) | b – Central Europe (CEU) | c – Southern Europe (SEU) |
| --- | --- | --- |
|  |  |  |

**Figure S22.** Scatter plots and regression lines for normalized anomalies, ${NorX}_{m,y}$ according to Equation (2), of runoff (R, Y-axis) versus soil moisture (SM, X-axis), for (a) Northern Europe (NEU), (b) Central Europe (CEU), and (c) Southern Europe (SEU) by using the internally consistent dataset.

| a – Northern Europe (NEU) | b – Central Europe (CEU) | c – Southern Europe (SEU) |
| --- | --- | --- |
|  |  |  |

**Figure S23.** Scatter plots and regression lines for normalized anomalies, ${NorX}_{m,y}$ according to Equation (2), of temperature (T, Y-axis) versus soil moisture (SM, X-axis), for (a) Northern Europe (NEU), (b) Central Europe (CEU), and (c) Southern Europe (SEU) by using the internally consistent dataset.

**Figure S24.** Coefficient of determination (r2) between normalized monthly anomalies, ${NorX}_{m,y}$ according to Equation (2), of evapotranspiration (ET), precipitation (P), runoff (R), and temperature (T) versus those of soil moisture (SM) with consideration of a backward 1-month shift (red), no time shift (green), and a forward 1-month shift (blue) between the variables. Results are presented for North Europe (NEU), Central Europe (CEU), and Southern Europe (SEU) based on the intermediate dataset (left three graphs) and the internally consistent dataset (right three graphs).

|  |  |  |
| --- | --- | --- |
|  |  |  |
|  |  |  |

**Figure S25.** The highest monthly coefficient of determination (r^2^) from the results for different temporal shifts (including no shift) of the normalized monthly anomalies$({NorX}_{m,y}$, according to Equation (2)) of runoff (R), precipitation (P), evapotranspiration (ET) and temperature versus those of soil moisture (SM) in (a,d,g) North Europe (NEU), (b,e,h) Central Europe (CEU), and (c,f,i) Southern Europe (SEU).
